# Genomic prediction of unordered categorical traits: an application to subpopulation assignment in German Warmblood horses

**DOI:** 10.1186/s12711-016-0192-2

**Published:** 2016-02-11

**Authors:** Claas Heuer, Christoph Scheel, Jens Tetens, Christa Kühn, Georg Thaller

**Affiliations:** Institute of Animal Breeding and Husbandry, University of Kiel, Hermann-Rodewald-Strasse 6, 24098 Kiel, Germany; Institute for Genome Biology, Leibniz Institute for Farm Animal Biology, Wilhelm-Stahl-Allee 2, 18196 Dummerstorf, Germany; Faculty of Agricultural and Environmental Sciences, University Rostock, Justus-von-Liebig-Weg 6, 18059 Rostock, Germany

## Abstract

**Background:**

Categorical traits without ordinal representation of classes do not qualify for threshold models. Alternatively, the multinomial problem can be assessed by a sequence of independent binary contrasts using schemes such as one-vs-all or one-vs-one. Class probabilities can be arrived at by normalization or pair-wise coupling strategies. We assessed the predictive ability of whole-genome regression models and support vector machines for the classification of horses into four German Warmblood breeds.

**Results:**

Prediction accuracies of leave-one-out cross-validation were high and ranged from 0.75 to 0.97 depending on the binary classifier and breeds incorporated in the training. An analysis of the population structure using eigenvectors of the genomic relationship matrix revealed clustering of individuals beyond the given breed labels. Admixture between two breeds became apparent which had substantial impact on the prediction accuracies between those two breeds and also influenced the contrasts between other breeds.

**Conclusions:**

Genomic prediction of unordered categorical traits was successfully applied to subpopulation assignment of German Warmblood horses. The applied methodology is a straightforward extension of existing binary threshold models for genomic prediction.

## Background

Categorical traits play an important role in livestock breeding. It is often possible to arrange K distinct classes of a particular trait into a meaningful order. Ordinal threshold models can then be applied by introducing K − 1 thresholds to the liability. However, if ordering classes is doubtful or impossible, those threshold models do not apply in a meaningful manner. This is the case when treating subpopulations or breeds (both terms are used interchangeably throughout the paper) as classes of the trait “subpopulation assignment”. Partitioning individuals into groups or subpopulations is elementary in livestock breeding and can be done in various ways based on geographical, phenotypical or ideational criteria.

In breeding organisations, the composition of a particular breed is subject to selection, migration and drift. Also, breed definitions and breeding goals change over time, making stringent permanent classification difficult. Different subpopulations of the German Warmblood horse have different breeding schemes, breeding value definitions and evaluations, geographical locations, ancestral breed compositions and migration policies. We expect those differences to be visible on the genomic scale using high-density single nucleotide polymorphisms (SNPs). The most fundamental metric to measure divergence between populations is the fixation index [1], which allows detection of differences in allele frequencies at a particular genomic locus between subpopulations. An aggregate of fixation indices over all available loci (e.g. SNPs) can be used as a measure of genetic distance between subpopulations.

At this point, it is important to distinguish between divergence between subpopulations caused by genetic drift and gene flow (demographic history) and actual selection, both of which will be reflected by the fixation index. When sequence data is available, this can be assessed by distinguishing between SNPs located in genic and nongenic regions (neutral markers) [2]. From a quantitative genetic perspective, estimating genetic variances and covariances of traits under selection between breeds can provide insights into the amount of genetic differentiation at quantitative trait loci (QTL). However, the trait under investigation (assignment to subpopulation) reflects both sources of divergence and does not allow a direct differentiation between them.

Genomic prediction is widely used for the prediction of quantitative and qualitative traits in livestock, plant breeding and human genetics. It has been shown, that regression on markers can pick up genomic relationships between individuals as well as linkage disequilibrium and co-segregation between markers in founders and descendants, respectively [3]. Breed composition is based on demographic history, migration policies and selection of quantitative/qualitative traits.

The aim of this study was to investigate the ability of whole-genome regression methods that are used for genomic selection, to discriminate between subpopulations. We did not seek to use genomic prediction methodology to cluster a given data set into subsets, but used predefined subpopulation assignments, which were assumed as fixed cluster indices, and assessed the predictive ability of various methods. However, as a consequence of this approach being applied to subpopulation categories, predictions of uncategorized individuals, based on the training of marker effects, can be used for subpopulation assignments or probabilistic assignments to several subpopulations. In addition, the discriminating ability of models based on genome-wide dense SNP panels can be used as a criterion for the divergence between breeds.

Genomic prediction of ordinal traits has been successfully applied in animal and plant breeding using either threshold models [4–7] or categorical discriminating machine learning techniques like support vector machines [7]. The prediction of unordered categorical traits involves several difficulties, which are addressed in the following section.

## Methods

### Dataset

The dataset included 917 stallions from four German Warmblood horse breeding populations that were part of a breed survey between 2005 and 2008, i.e. Hanoverian, Holsteiner, Oldenburger and Trakehner. The animals were genotyped with the Illumina Equine-SNP^®^ BeadChip that contains 54,602 SNPs. Genotyping results for autosomal SNPs only were filtered based on Illumina’s GenCall score applying a threshold of 0.15, which resulted in a mean call rate higher than 0.98. Furthermore, markers with a minor allele frequency less than 0.05, and a genotyping rate of at least 90 % were discarded. The final set comprised 44,159 SNPs. Before excluding SNPs based on the described criteria, we analysed each SNP for opposing fixation of certain alleles between the breeds. This should have allowed prediction of subpopulation assignment based only on those SNPs. However, none of the SNPs showed opposing fixation of alleles.

The pedigree consisted of 270 unique sires and 876 unique dams, yielding a pedigree size of 2061 individuals that are summarized in Table 1. It should be noted here that the analysis of the different breeds was based on the sample of available stallions and might not appropriately reflect the real breed compositions.

**Table 1:** Overview of the analysed subpopulations

Subpopulation	Numeric indicator	Individuals	Sires	Dams
Hanoverian	1	306	95	296
Holsteiner	2	348	98	322
Oldenburger	3	219	95	216
Trakehner	4	44	33	43

### Genomic prediction of unordered categorical traits

When investigating categorical traits, for which the different classes cannot be arranged in a meaningful order, the distinct categories have to be treated as K independent classes termed or labelled ${C_k}$, where *k* is a value between 1 and K. In that case, the use of ordinal threshold models is questionable. We have treated the multiple class prediction problem as one that can be represented by binary contrasts between two or more classes. In logistic regression, the multinomial case is dealt with by conducting K − 1 binary contrasts against a base line class. Several schemes are possible to combine a collection of binary classifiers into a single multinomial classifier. The main difficulty is to define the scheme in a consistent way and to guarantee a unique classification, since more than one binary classifier might claim the same data for different classes or none of the binary classifiers might claim the data at all. A customary scheme, also used here, is the one-vs-all (OVA), where each of the K classes is paired with the complement of samples from the K − 1 other classes. This will almost always be unbalanced, since each binary classifier usually will see a larger number of negative samples consisting of the $K-1$ classes in the complement of its positive class. The second customary scheme is the one-vs-one (OVO), where each possible $K(K-1)/2$ pairing of classes is presented to a binary classifier. The classification of the data into the different classes is then decided by ’voting’. Unobservable data $\mathbf {x}$, where $\mathbf {x}$ is a vector with D features (e.g. SNP covariates), can be classified according to the class that has most often been predicted by the binary classifiers.

In case of a probabilistic classifier such as the probit threshold model used here, one would, instead of working with binary classifications only, also seek to obtain a full *K*-dimensional vector of posterior predictive probability assignments:

$$\begin{aligned} \mathbf {p} = (p_1,...,p_K) = \mathbf {p(x)}, \quad \text {where } p_k = Pr(C_k | \mathbf {x}) :\equiv Pr(class(\mathbf {x})=C_k|\mathbf {x}), \end{aligned}$$

before committing to a class by picking the class having the maximal probability for the individual $\mathbf {x}$. Since only the conditional probability estimates from the binary machines are available, the task to consistently assign probabilities fundamentally hinges on these:

$$\begin{aligned} \hat{p}_{kk'} \sim p_{kk'} = Pr(class(\mathbf {x})=k|class(\mathbf {x})\in \{C_k,C_{k'}\}, \mathbf {x}), \end{aligned}$$

where the prime in $k'$ represents one of the binary classification pairings to $C_k$ as given by the scheme employed. In case of the OVA scheme, this is relatively straightforward:

$$\begin{aligned} Pr(C_k |\mathbf {x}) = \frac{Pr(C_k|class(\mathbf {x}) \in \{C_k,C_{k'}\},\mathbf {x} )}{\sum _{l=1}^{K} Pr(C_l|class(\mathbf {x}) \in \{C_l,C_{l'}\}, \mathbf {x})}. \end{aligned}$$

In the OVO scheme, it is certainly possible to implement class probabilities from the voting scheme’s $\{0,1\}$ valued table i.e.:

$$\begin{aligned} p_k = \frac{2}{K(K-1)} \sum _{k',k'\ne k} c_{kk'}, \quad \text {where} \quad c_{kk'} = 1_{\{ p_{kk'} > p_{k'k}\}}, \end{aligned}$$

as a counting measure, but this would be based on already predicted class assignments. To retain the binary classifiers probabilities for estimating $\mathbf {p}$, we therefore followed an alternative pair-wise coupling approach given in Wu et al. [8], where the posterior predictive probabilities are directly estimated from those of the binary classifiers. The known pair-wise probability estimates $p_{kk'}$ should, even if approximately, require the unknown class probabilities $p_k$ and $p_{k'}$ to satisfy the equation:

1 $$\begin{aligned} p_{kk'} = \frac{p_k}{(p_k+p_{k'})}. \end{aligned}$$

Fixing each *k* and summing over the complementary $K-1$ classes $k'$ results in the determining system:

$$\begin{aligned} p_k = \sum _{k',k' \ne k} \frac{p_k+p_{k'}}{K-1} p_{kk'} \quad \text {with constraints} \quad p_k > 0,\quad \sum _{l=1}^{K} p_l = 1, \end{aligned}$$

for the probability vector $\mathbf {p}=(p_1,...,p_K)$ to be obtained. This is recast as a fixed point problem in [8]. Thus, under the constraints given above still, $\mathbf {p}$ is to solve the following fixed point equation:

$$\begin{aligned} \mathbf {p}=\mathbf {Qp} \qquad \text {with} \quad Q_{kk'} = \frac{1}{K-1} \left\{ \begin{array}{l l} p_{kk'} &{} \quad \text {if }k \ne k' \\ \sum\nolimits_{l \ne k}^{K} p_{kl}&{} \quad \text {if }k = k' \end{array} \right. . \end{aligned}$$

Based on the Markov property of *Q*, it is then shown that this yields a unique solution although Eq. (1) is overdetermined, and it is argued that this solution is a proper model to build multi-class probabilities from binary ones.

#### Threshold model

Bayesian regression models for binary contrasts have been applied using a threshold model. The conditional success probability for the vector of observed binary responses (breed assignments) $\mathbf {y}$ can be written as:

2 $$\begin{aligned} Pr(\mathbf {y}=1|\varvec{\eta }) = \Phi (\varvec{\eta } - \delta ), \end{aligned}$$

where $\varvec{\eta } = \mathbf {Wb} + \mathbf {Zu}$ represents a linear predictor for the liability that is assumed to give rise to the measured phenotypes:

$$\begin{aligned} \mathbf {l}=\varvec{\eta } + \mathbf {e}, \end{aligned}$$

where $\mathbf {e}$ is assumed to follow $\sim N(\mathbf {0},\mathbf {I})$. $\mathbf {W}$ and $\mathbf {Z}$ are known incidence matrices for fixed and random effects with $\mathbf {b}$ and $\mathbf {u}$ being their respective solution vectors.

The linear predictor is mapped to the expectation of $\mathbf {y}$ through the probit link function $\Phi$, the standard normal cumulative distribution function. In our case with only two classes in the response vector, $\delta$ is a single threshold parameter. The predicted probabilities from the threshold models can be used to obtain class probabilities as discussed in the previous section.

#### Estimation of multinomial breeding values

The computation of multinomial predictors based on binary contrasts can also be used to estimate multinomial breeding values expressed as expected fractions of offspring in the different categories. Considering the probit threshold model described above and a OVA scheme, a base population using the allele frequencies of the marker covariates ($\mathbf {f}$) in, e.g., founders, can be defined. Let $\mathbf {z_n}$ be a vector of marker covariates for selection candidate *n*, $\mathbf {u}_k$ a vector of marker effects for class *k*, $\overline{\mathbf{w}}$ a vector of average fixed effect coefficients and $\mathbf {b}_k$ a vector of fixed effects for class *k*. $p_{n_k}$ contains the expected fraction of offspring in class *k* for individual *n* averaged over the fixed effects and can be obtained as:

$$\begin{aligned} p_{n_k} = \frac{\Phi ( \overline{\mathbf{w}}'\mathbf {b}_k + 0.5((\mathbf {z_n} - 2\mathbf {f})'\mathbf {u}_k) - \delta _k)}{\sum _{l=1}^{K}\Phi (\overline{\mathbf{w}}'\mathbf {b}_l + 0.5((\mathbf {z_n} - 2\mathbf {f})'\mathbf {u}_l) - \delta _l)}, \end{aligned}$$

where $\delta _k$ is the threshold for the *k*th binary OVA model. Deviations of the offspring from the population means can be computed as $\mathbf {g}_n = \mathbf {p}_{n} - \mathbf {p}_{0}$, where $\mathbf {p}_{0}$ is a vector of the class fractions in the base population.

#### Bayesian regression models

Genomic prediction for the binary traits has been conducted using the general model given in Eq. (2) but using two different models for the liability. Those models differ in their prior distribution for the marker effects $\mathbf {u}$. The most prominent model, Ridge Regression (or equivalently GBLUP), assumes a Gaussian prior on $\mathbf {u}$ with a common variance component. In addition, we also employed BayesA [9], which effectively assumes a t-prior on $\mathbf {u}$ [10].

#### Support vector machines

Support vector machines (SVM) [11] are a supervised linear model for binary classification. A simple linear model or hypothesis is a function of the form:

$$\begin{aligned} y(\mathbf {x}) = \beta _0 + \mathbf {x'}\varvec{\beta } = \beta _0 + \langle \mathbf {x} |\varvec{\beta } \rangle , \end{aligned}$$

where $\langle .|. \rangle$ denotes the standard scalar product, the argument $\mathbf {x}$ is a column vector of features (e.g. one of a marker or design matrices’ rows), $\beta _0$ is an intercept and $\varvec{\beta }$ is a *D* dimensional vector of weights to be determined during a training procedure. Such a function, given that $\varvec{\beta }\ne 0$, defines a hyperplane on its domain (the feature space) by $y^{-1}(c)$ for any real constant c, the subspace of points $\mathbf {x}$ satisfying the equation $y(\mathbf {x})=c$. This geometrically splits the feature space in two halves, and the hyperplane is to become the so-called separating hyperplane or decision boundary for a binary classification problem. Given paired data $(\mathbf {x}_n,c_n)_{1 \le n \le N}$, where N is the number of samples and $c_n$ are realized class labels with values within $\{-1,1\}$, a support vector machine seeks to adjust or learn $\varvec{\beta }$ such that the hyperplane given by $y(\mathbf {x})=0$ separates the classes according to their labeling. A dataset/labeling which admits this is called linearly separable. In this case, usually an infinite number of such separating planes exists, and the SVM will find a maximum margin solution. It is the plane that has the same maximally possible orthogonal distance to either of the classes. The data points touching the margin are called the support vectors, as these serve to define the separating plane and the margin. If the dataset is intertwined and not linearly separable, the margin serves as slack area (i.e. a volume in feature space), where misclassification is allowed.

Having established the basic binary SVM classifier, there are two important extensions to be made: a scheme for concatenating binary classifiers, such as described above, can be set up to build a single multinomial classifier, and some (nonlinear) feature transformation can be carried out before using the basic SVM to accommodate for nonlinearities in the data. A feature transformation is an arbitrary (but somewhat 'reasonable') mapping $\phi$ of the data into some other feature space. The linear model then follows the form:

$$\begin{aligned} y(\mathbf {x}) = \beta _0 + \phi (\mathbf {x})'\varvec{\beta }= \beta _0 + \langle \phi (\mathbf {x}) | \varvec{\beta } \rangle . \end{aligned}$$

Frequently, the data is mapped to a higher dimensional space under $\phi$. The embedding of the data in a higher dimensional space increases the chances that the image of the data under $\phi$, is linearly separable. An example for a feature space transformation is to let $\phi$ have the components $\phi _n= \langle \mathbf {x}_n , . \rangle$, where $\mathbf {x}_n$ might represent marker covariates for training. This is in essence a genomic relationship matrix. Here, the mapping $\phi$ depends on training data, and the dimension depends on the number of samples (individuals) available. The genomic relationship matrix can also be viewed as stemming from a kernel.

A kernel is usually given by a bivariate function *K* that takes the form:

3 $$\begin{aligned} K(\mathbf {x}_1,\mathbf {x}_2) = \langle \phi (\mathbf {x}_1)|\phi (\mathbf {x}_2)\rangle _H, \end{aligned}$$

for some feature mapping $\phi$ and some Hilbert space *H* with scalar product $\langle .| . \rangle _H$. Note that it is sufficient to know and evaluate an admissible (Mercer’s condition) *K* on pairs of data, while, conversely, the RHS of Eq. (3) defines a kernel in any case. The standard genomic relationship matrix emerges as a special case [12], it is the kernel from letting $\phi =id$, the identity mapping [$\phi (\mathbf {x}) = \mathbf {x}$].

This also reduces the dimension to the number of samples available, which, in general, will be smaller than the dimension of the marker covariates. Therefore, in our analysis, we concentrated on the original marker representation to be used with the support vector machine.

#### Measuring prediction accuracy

Prediction accuracy was measured in two ways, depending on the classification rule via *leave-one-out* cross-validation. Although this scheme is computationally very expensive, it was chosen because of the unbalanced representation of the different classes in our dataset and the very low numbers observed for the Trakehner class. The *leave-one-out* scheme ensures the highest possible absolute number of training animals while reflecting the structure of the data in a very similar fashion in each run. We used discrete classification from the support vector machines directly, while assigning the class with highest probability from the Bayesian regression models and measured the prediction accuracy in the discrete case as:

$$\begin{aligned} \text {Accuracy} = \frac{\text {Number of correct classifications}}{\text {Total number of classifications}}. \end{aligned}$$

For the regression models, another metric that sums up squared differences between actual class category and predicted probabilities, was also used (Brier Score [6, 13, 14]):

$$\begin{aligned} \text {Brier Score} = N^{-1}\sum _{i=1}^{N}\sum _{j=1}^{K}\bigl (p_{ij} - [y_i = y_{ij}]\bigr )^2, \end{aligned}$$

where $y_{ij}$ is an indicator that takes a value of 1 if the observed value $y_i$ is *j* and 0 otherwise and $p_{ij}$ is the predicted probability for individual *i* for class *j*. This score ranges from 0 to 2, with lower values indicating higher accuracy. In order to make inference about the prediction accuracy of a given model, it is necessary to compare it to a base line model for which we assume that the probability for any individual and class is $p_{ij} = K^{-1}$, yielding equal probabilities for the assignment of any class. The relative performance of the models is reported as one minus the fraction of the Brier Score to the Brier Score of the base line model (*sBS*). Hence, an *sBS* value of 0 indicates that the model predicts no better than random assignments of individuals to classes with uniform probabilities. A value close to 1 indicates perfect prediction. This is only slightly different from the definition given in Steyerberg et al. [15], where the base line model uses the population frequencies of the classes, contrary to the uniform frequencies in the metric used here.

### Population structure

#### Definition of subpopulations

Subpopulations or breeds can be defined in various ways and this definition has an impact on the interpretation of results. The general assumption of our linear model is that the response, which is the assignment of an individual to a subpopulation, is measured without error. The absence of measurement error can only be assessed with respect to the breed definition. One possible definition might be based on a combination of phenotypes that unambiguously defines the breed. In this case, the phenotype “subpopulation assignment” is a linear or nonlinear combination of measurable phenotypes and will therefore be confounded with all phenotypes that are being used to define the particular subpopulations.

In the case of the present data set, the animals were assigned to the different breeds by their corresponding breeding organizations. Those assignments are variable in their restrictiveness, since one organization might be more open to migration than others. In other words, the phenotype “subpopulation assignment” was not identically defined across breeding organisations.

Actually, the Trakehner horse breed has been partially closed since 1732 and only introgression of Thoroughbreds and Arab horses are allowed. Their total gene contribution has been estimated at 34 % [16]. The Holsteiner horse breed has historically also been influenced by Thoroughbred and Arab horse breed introgression as well as by the French Selle Francais that has had considerable influence. Total contributions of approximately 26 and 16 %, respectively, have been estimated for these breeds [17]. The actual Holsteiner breed policy, however, restricts the use of foreign breeding animals [http://www.holsteiner-verband.de/front_content.php?idcat=408]. The Hanoverian breeding policy can be regarded as more open than that for the aforementioned populations. The Hanoverian breed has also been influenced by several waves of Thoroughbred introgression, but also by the Trakehner horse breed, which has been extensively used after World War II, and also by the Holsteiner breed since 1993, but to a lesser extent in the context of the Hanoverian Jumper Breeding program. It has been estimated that approximately half of the Hanoverian gene pool is attributable to original Hanoverian founder animals [18]. Finally, the Oldenburger association operates the most open breeding program. According to its statutes, a wide variety of other breeds and populations is allowed [http://www.oldenburger-pferde.net/upload/Satzung_Stand_Juni_2014__korrigierte_Version].

#### Analysis of population structure

In order to assess the population structure of the data set, a principal component analysis was conducted using the realized genomic relationships based on the available markers [19]. Genomic relationships across breeds were computed according to VanRaden [20] as: $\mathbf {G} = \frac{\mathbf {MM}'}{\sum _{i=1}^{m} 2p_i(1-p_i)}$, where $\mathbf {M}$ is a matrix containing centered coefficients of gene content for the 44,159 SNPs. In order to investigate potential clustering of the data, a scatterplot of the first two eigenvectors of the decomposition $\mathbf {G} = \mathbf {UDU}'$ was visually inspected. In addition, we performed an eigenvector analysis of the genomic relationship matrices between pairs of breeds $\{kk'\}$, which corresponds to the approach of Janss et al. [21] for quantitative traits.

A threshold model of the form given in Eq. (2) was assumed, with $\mathbf {Z}$ being a random effects design matrix with columns representing the eigenvectors of $\mathbf {G}_{kk'}$. The prior assumption on the vector $\mathbf {u}$ of random effects (solution to the regression on the eigenvectors in $\mathbf {Z}$) was a point mass at zero plus a Gaussian slab. This basic variable selection model corresponds to the approach of Kua and Mallick [22] and Habier et al. [23] (BayesC$\pi$).

Janss et al. [21] used the proportion of explained variance by an eigenvector *j* times the estimated regression coefficients $\alpha _j$ to the total sampling variance of the genetic values $\mathbf {g}$ (=$\mathbf {U\varvec{\alpha }}$) for estimating the importance of individual eigenvectors. By defining a threshold of explained variance similar to that of Fernando and Garrick [24], eigenvectors that contribute significantly to the sampling variance in $\mathbf {g}$ can be identified. The difference here is that we use the inclusion probabilities of the eigenvectors in the BayesC$\pi$ model directly to make inference about the importance of certain eigenvectors. A similar approach for genome-wide association studies was proposed by Fernando et al. [25], where the indicator variable for all markers in a specified window is used in a BayesC model for computing the posterior probability of an association for that window.

We report the posterior mean of the proportion of eigenvectors with non zero effect (*pEV*) as a measurement of model complexity. Higher values of *pEV* indicate a more complex relationship between two breeds, hence less discriminative power of single eigenvectors. An alternative would be to treat one eigenvector at a time as the response variable and perform an analysis of variance using the known class labels as the explanatory factor variable as in Patterson et al. [19], but this was not investigated here.

The power of the available markers to discriminate between the breeds was assessed through cross-validation using the multinomial prediction scheme described above. Furthermore, we estimated pedigree- and marker-based heritabilities between pairs of breeds, using either $\mathbf {A}_{kk'}$ or $\mathbf {G}_{kk'}$ as relationship matrix in an animal model, for the trait “subpopulation assignment” using Gibbs sampling. The heritability can then be computed as: $h_{Ped/Marker}^2 = \sigma _{a_{Ped/Marker}}^2 / (\sigma _{a_{Ped/Marker}}^2 + \sigma _{e}^2)$, where $\sigma _{a_{Ped/Marker}}^2$ is the additive genetic or genomic variance component, respectively. The residual variance component ($\sigma _{e}^2$) is set to 1 (probit model).

In addition, marker-wise fixation indices ($F_\text {ST}$) between pairs of and across all breeds were estimated. This quantity is defined as the ratio between variation in allele frequencies between breeds ($\sigma _{\alpha }^2$) and the expected heterozygosity ($\sigma _{\epsilon }^2$) at the locus ($F_\text {ST} = \frac{\sigma _{\alpha }^2}{\sigma _{\epsilon }^2}$). Performing an analysis of variances to test for significant differences in allele frequencies between breeds can be used to identify loci that contribute to the discrimination between breeds. We estimated $F_\text {ST}$ marker-wise using a linear mixed model by treating gene content as the response variable and subpopulations as random effects assuming $var(\varvec{\alpha }) = MVN(0,\mathbf {I}\sigma _{\alpha }^2)$ [26], with $\mathbf {I}$ being an identity matrix of order *K*. It should be noted that the traditional $F_\text {ST}$ value assumes Hardy-Weinberg equilibrium (HWE) for any marker, since it uses the expected heterozygosity as a measure of sampling variance for the markers. We report this measurement when comparing the four different subpopulations pair-wise, since the estimation of the variance of a random effect with only two levels requires introducing some sort of prior knowledge.

However, for the whole population, the global $F_\text {ST}$ values were estimated in a mixed model analysis and we report the ratio of the variance in allele frequencies between the subpopulations and the total model variance. Such estimation of $F_\text {ST}$ has a strong connection to the recently proposed method of Forneris et al. [27] based on the initial approach of Gengler et al. [28], for which they assume that $var(\varvec{\alpha }) = MVN(0,\mathbf {A}\sigma _{\alpha }^2)$, with $\mathbf {A}$ being the numerator relationship matrix. In their approach, they use departures of the estimated heritability from one as a measure of genotyping error for the loci.

#### Software

All evaluations were performed in R [29]. The Bayesian threshold models have been implemented using BGLR [30]. Classification using SVM was done with e1071 [31], which has libsvm [32] as a backend. We used default prior specifications (BGLR) and tuning parameters (e1071). Population-wide $F_\text {ST}$ values were estimated using Asreml-R 3.0 [33]. The BayesC$\pi$ threshold model for the eigenvector analysis was implemented in JAGS [34] using the rjags package [35]. For the genomic prediction regression models, the Gibbs sampler was run for 30,000 iterations (15,000 burn-in). For the estimation of heritabilities and *pEV* using BGLR and JAGS, respectively, the number of iterations was 60,000 while discarding the first 20,000 as burn-in.

## Results and discussion

### Population structure

**Fig. 1:**
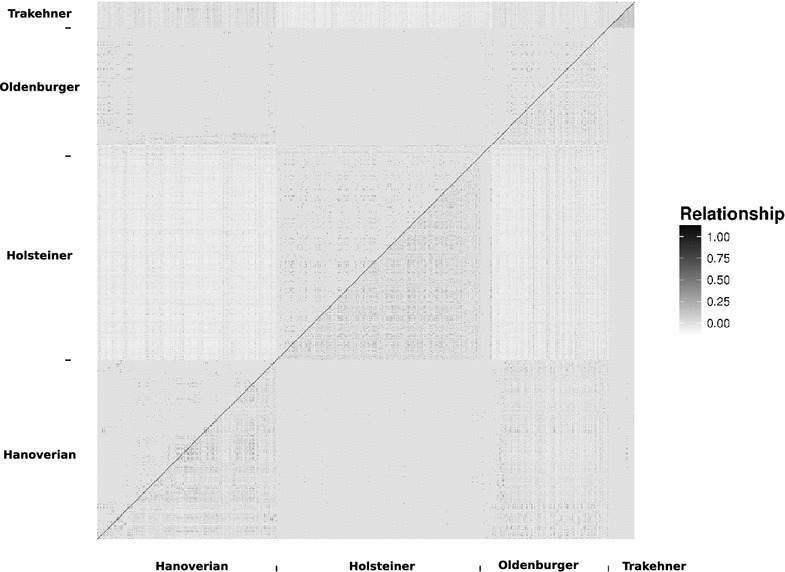
Pedigree-derived and marker-derived additive relationship matrices arranged by subpopulations. The *upper and lower triangular matrices represent the pedigree-derived and marker-derived additive relationships, respectively. Diagonals are set to 1*

An overview of the breeds under investigation is in Table 1. For the 917 animals in the data set, 264 stallions were used as sires, of which 46 were used in more than one breed. Most significantly, Hanoverian and Oldenburger populations shared 34 sires which resulted in strong half-sib structures within and between breeds. The additive genetic relationship structure is illustrated in the upper triangular part of Fig. 1. The genomic relationships show the high within-breed relationships based on the observed marker genotypes. A strong connection between the Hanoverians and Oldenburger breeds beyond the half-sib structures becomes apparent, while the Holsteiner and Trakehner breeds show very little genomic relationship with other breeds besides the half-sibs. This suggests that those breeds introduce only very few foreign sires into their breed. Although there is a lot of exchange of sires between the breeds, for the Holsteiner and Trakehner breeds, it is mainly unidirectional.

Averages over the additive genetic and genomic relationships are in Table 2. Since the genomic relationship matrix is centered based on the allele frequencies of the whole sample, there are negative values that indicate below average relationships in this sample of individuals. The Holsteiner breed shows below average relationships with all other breeds while the Hanoverian breed has positive average relationships with the Oldenburger and Trakehner breeds. Average genomic relationships (and standard deviations thereof) in distinct breeds are equal to 0.022 (0.055) for Hanoverians, 0.037 (0.06) for Holsteiner, 0.0116 (0.054) for Oldenburger and 0.10 (0.053) for Trakehner. The highest average pedigree relationship was observed for the Hanoverian and Oldenburger breeds (0.00269), while the average pedigree relationships within those breeds were equal to 0.0082 and 0.0056, respectively.

**Table 2:** Measures of relationship and separability between subpopulations

Base	A	G	${F_{st}}$	${h_{Ped}^2}$
12	0.0002 (0.0075)	−0.0290 (0.0321)	0.0151 (0.0196)	0.9809 (0.0158)
13	0.0029 (0.0269)	0.0085 (0.0447)	0.0029 (0.0040)	0.9179 (0.0675)
14	0.0008 (0.0143)	0.0123 (0.0334)	0.0070 (0.0100)	0.8444 (0.0779)
23	0.0003 (0.0084)	−0.0176 (0.0377)	0.0109 (0.0147)	0.9442 (0.0269)
24	0.0000 (0.0000)	−0.0269 (0.0252)	0.0117 (0.0176)	0.9207 (0.0700)
34	0.0001 (0.0057)	0.0003 (0.0262)	0.0097 (0.0137)	0.7967 (0.1376)

Base	${h_{Marker}^2}$	*pEV*	nEV	CV-Accuracy
12	0.9575 (0.0180)	0.0133 (0.0056)	8.6981 (3.6581)	0.9832
13	0.8828 (0.0546)	0.0480 (0.0227)	25.1528 (11.8885)	0.7581
14	0.9233 (0.0417)	0.0122 (0.0071)	4.2579 (2.4701)	0.9457
23	0.9441 (0.0292)	0.0209 (0.0086)	11.8532 (4.8888)	0.9418
24	0.9214 (0.0532)	0.0061 (0.0043)	2.3697 (1.6806)	0.9974
34	0.9253 (0.0556)	0.0176 (0.0100)	4.6038 (2.6294)	0.9544

The averages (standard deviations) of pair-wise additive genetic relationship (A), genomic relationship (G), average $F_\text {ST}$ values of all markers, pedigree-based heritability on the liability scale ($h_{Ped}^2$), marker-based heritability on the liability scale ($h_{Marker}^2$) for the trait “subpopulation assignment”, percentage (*pEV*) and number (nEV) of eigenvectors included in the variable selection threshold model and the prediction accuracy of subpopulation assignment (CV-Accuracy) of a leave-one-out cross-validation using SVM. Base indicates the classes used in the pairwise contrasts. Numeric indicators for subpopulations are: *1* Hanoverian, *2* Holsteiner, *3* Oldenburger, *4* Trakehner

The absolute and relative differences in the mean values of these measurements were small, while average $F_\text {ST}$ values were more contrasted, although on a lower scale. The Hanoverians and Oldenburger breeds had by far the lowest mean $F_\text {ST}$ values (0.0029), which means that averaged over all markers, the variance in allele frequencies between these two breeds was small compared to the overall (expected) variance (heterozygosity). The magnitude of $F_\text {ST}$ values was most marked between the Holsteiner and any of the other breeds with a maximum value of 0.0151 against the Hanoverian breed. $F_\text {ST}$ values across all breeds ranged from 0.00 to 0.513, with an average of 0.046. The summary $F_\text {ST}$ statistics were similar across all chromosomes.

#### Eigenvector analysis

**Fig. 2:**
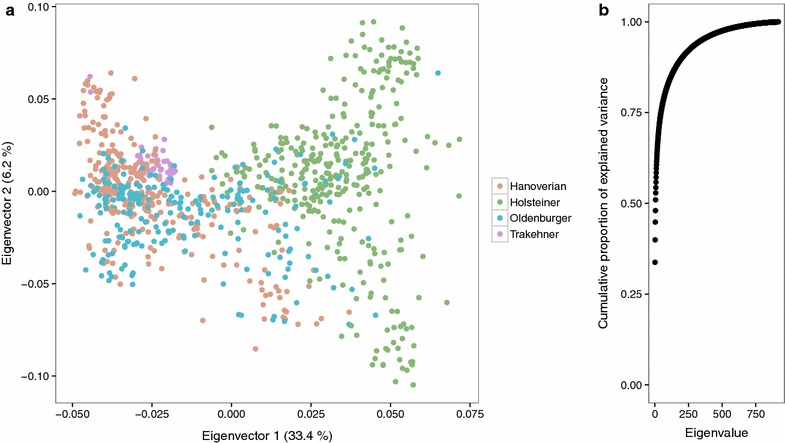
Scatterplot of the first two eigenvectors of the genomic relationship matrix (**a**) and cumulative proportion of explained variance by eigenvalues in decreasing order (**b**)

The first two eigenvectors of the genomic relationship matrix of all individuals in the data set were used to visually inspect the clustering of individuals according to their assigned breeds (Fig. 2a). The Holsteiner breed clearly clustered separately from the other breeds along the first axis. However, substructure of this population was also apparent along the second axis. A majority of the Hanoverians and Oldenburger animals formed a cluster along both axes, while the Oldenburger individuals were broadly spread along the first axis into the Holsteiner population. The Trakehner animals clustered horizontally separately from the Holsteiner cluster at the border of the Hanoverian cluster. The cumulative proportion of explained variance by the eigenvectors is in Fig. 2b, and reveals that the first five eigenvectors already explain more than 50 % of the variation in the data. This supports the visual result of the strong discriminative ability of the first two eigenvectors. The eigenvector clustering shows significant overlap between breeds (e.g. Hanoverians and Trakehner), while subclusters within breeds were apparent. Hence, the data could be divided into different and more diverse clusters than the four breed labels. This finding was also supported by an evaluation of the data set using fastSTRUCTURE [36], which proposed a clustering into 15 populations (data not shown).

**Fig. 3:**
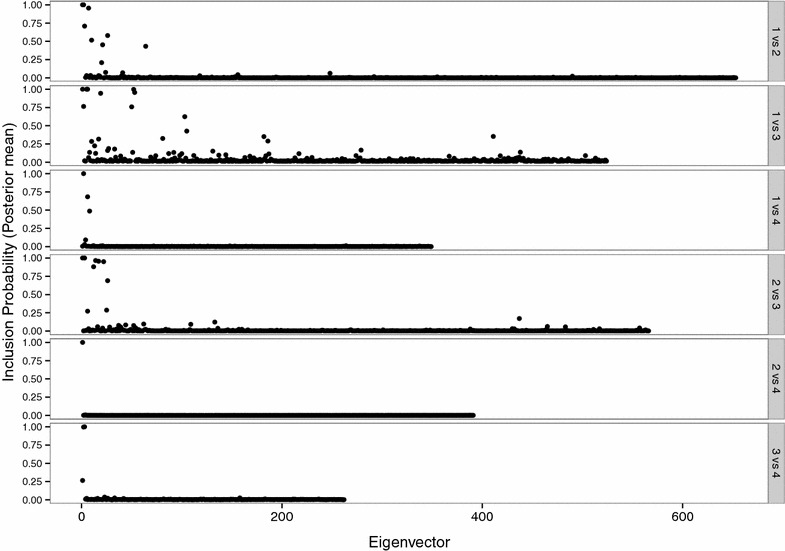
Inclusion probabilities of eigenvectors by pair-wise contrasts. Results show posterior means of the inclusion probabilities for eigenvectors that were obtained from pair-wise genomic relationship matrices and fitted as random effects in the BayesC$\pi$ threshold model. The pair-wise response variable was “subpopulation assignment”. Numeric indicators for subpopulations are as follows: *1 *Hanoverian, *2* Holsteiner, *3* Oldenburger, *4* Trakehner

In addition to the visual inspection, we used eigenvectors to estimate two additional metrics that are in Table 2. *pEV* is a measure of model complexity using the eigenvectors of the genomic relationship matrix between two breeds ($\mathbf {G}_{kk'}$) as random effects in the BayesC$\pi$ threshold model. If two populations differ at most of the SNPs, we would not expect more than one eigenvector to be necessary to distinguish between them [19]. Hence, *pEV* and the number of eigenvectors with non-zero effects ($nEV = pEV \times (N-1)$, with *N* being the number of individuals in the sample of the two breeds), serve as a measure of divergence between the two breeds, with higher values of *pEV* indicating lower divergence. The posterior means of *pEV* ranged from 0.0061 (Holsteiner vs. Trakehner) to 0.048 (Hanoverian vs. Oldenburger), resulting in on average 2.4 and 25.15 eigenvectors included in the models, respectively. The individual inclusion probabilities of the eigenvectors in the pair-wise contrasts are in Fig. 3. These estimates support the results from the pair-wise cross-validation (CV-Accuracy, Table 2) but also suggest that, except for the Holsteiner and Trakehner pair of breeds, no pair of breeds formed distinct clusters. This result corresponds well to the breeding policies described earlier.

In addition to the eigenvector analysis used here, a wide range of methods exists to cluster datasets into subgroups and assign individuals to these. Besides distance measures used in k-means clustering or discriminative analysis, model-based approaches are frequently assessed. In particular, Pritchard et al. [37] and Raj et al. [36] used the aggregate of available marker genotypes to cluster individuals based on HWE and linkage equilibrium assumptions, while discriminating between the populations based on departures from both these metrics.

### Genomic prediction of subpopulation assignment

Besides the measurements already described in Table 2, three additional estimates, that quantify the ability of the available SNPs to discriminate between pairs of breeds, were investigated. These are inversely related to distance measures, which results in a higher discriminative ability of the SNPs and thus indicates that the distance between breeds is greater. Heritabilities on the liability scale of subpopulation assignment were estimated using either the numerator or genomic relationship matrix. They can be interpreted as the proportion of phenotypic variance (sum of the genetic and environmental variances) explained by the regression on the pedigree or marker covariates on the liability scale. This makes it necessary to define environmental (residual) variance in the case of subpopulation assignments. Assuming that the available sample of markers covers the genetic diversity across the whole genome between the breeds, there are two non-genetic sources of variance. First, error is introduced by the assignment of the different classes to breeds, hence phenotype measurement errors. Second, non-genetic or lowly heritable criteria are involved in the assignment of an individual to a subpopulation. In both cases, a heritability lower than 1 indicates non-genetic sources of variation for the phenotype “subpopulation assignment”. This quantity was estimated on the whole data set, hence it does not directly allow inference about the predictive ability. In general, pair-wise marker heritabilities were high, ranging from 0.88 (Hanoverians and Oldenburger animals) to 0.96 (Hanoverian and Holsteiner animals). The pedigree-based heritabilities ranged from 0.80 (Oldenburger and Trakehner) to 0.98 (Hanoverian and Holsteiner). Large differences between both relationship estimates are observed between Hanoverian and Trakehner and between Oldenburger and Trakehner animals together with larger standard deviations for $h_{Ped}^2$. Generally, the pedigree relationship matrix captures more recent relationships, which are particularly significant in the two-generation pedigree used here, while the markers capture more ancient relationships [38]. We would expect heritabilities of 0 for random class assignments, which would mean that all the variation in the phenotype is environmental or indeed random.

Since it may not be possible to generalize inferences without validation data [39], we used another metric based on pair-wise leave-one-out cross-validation using an SVM as binary classifier (See Table 2 CV-Accuracy). Resulting accuracies ranged from 0.76 (Hanoverians vs. Oldenburger animals) to 0.99 (Holsteiner vs. Trakehner animals) and reflect to which extent the SVM was able to predict unobserved data by learning marker weights in training data. Results using a Ridge Regression threshold model as the binary classifier were very similar (data not shown). Thus, it is obvious that there is strong predictive ability of the markers between any pair of breeds except for the Holsteiner and Oldenburger breeds. For these two breeds, prediction accuracy was higher than 0.5 (base line random assignment) but clearly lower than the accuracies achieved in the other pair-wise contrasts.

#### Prediction accuracies for the whole dataset

The results of the leave-one-out cross-validation are in Table 3. As binary classifiers, we used a Ridge Regression threshold model using all available markers and an untuned SVM. The Ridge Regression model was run using two coupling schemes, namely one-vs-one and one-vs-all. Accuracies for this model were achieved by assigning the class with the highest probability. For the SVM, only the one-vs-one scheme was used. The alternative threshold model using BayesA resulted in almost identical predictions as those obtained with Ridge Regression and are not included in Table 3. Furthermore, we employed five different base classes in the training data set, in order to identify classes that might enhance or decrease prediction accuracies. This was done by excluding one breed from the analysis entirely and estimating the prediction accuracies in the remaining breeds.

**Table 3:** Prediction accuracies and scaled Brier scores for subpopulation assignment

Base	Contrast	Accuracy					sBS				
		1	2	3	4	Total	1	2	3	4	Total
Ridge	Regression										
1234	One-vs-one	0.97	0.99	0.00	0.86	0.74	0.86	0.97	−0.43	0.66	0.58
1234	One-vs-all	0.86	1.00	0.54	0.86	0.84	0.69	0.98	0.18	0.68	0.68
234	One-vs-one		1.00	0.87	0.75	0.93		0.97	0.72	0.59	0.85
234	One-vs-all		1.00	0.87	0.93	0.95		0.98	0.70	0.77	0.87
134	One-vs-one	0.85		0.66	0.64	0.76	0.66		0.31	0.36	0.50
134	One-vs-all	0.85		0.67	0.82	0.78	0.66		0.32	0.59	0.52
124	One-vs-one	0.97	1.00		0.66	0.96	0.93	0.99		0.38	0.92
124	One-vs-all	0.97	1.00		0.80	0.97	0.92	0.99		0.61	0.94
123	One-vs-one	0.84	1.00	0.54		0.83	0.65	0.96	0.09		0.63
123	One-vs-all	0.85	1.00	0.53		0.83	0.65	0.97	0.07		0.63
SVM											
1234	One-vs-one	0.86	1.00	0.50	0.59	0.81					
234	One-vs-one		1.00	0.85	0.73	0.93					
134	One-vs-one	0.87		0.62	0.59	0.75					
124	One-vs-one	0.96	1.00		0.59	0.96					
123	One-vs-one	0.86	1.00	0.50		0.82					

The proportion of correct classifications to all classifications (Accuracy) and the scaled Brier Scores (sBS) per class and over all samples (Total) for the classification of subpopulation assignment using leave-one-out cross-validation. Base indicates the classes that were included in training and testing. Numeric indicators for subpopulations are:* 1* Hanoverian,* 2* Holsteiner,* 3* Oldenburger,* 4* Trakehner

In general, prediction accuracies were high and ranged from 0.59 to 0.97 across the whole data set. Using all classes for training results in intermediate overall prediction accuracies. OVO and OVA Ridge Regression yielded accuracies of 0.74 and 0.84, respectively, while the SVM reached an accuracy of 0.81. More interesting are the individual accuracies for the different subpopulations. In the OVO Ridge Regression, the Holsteiner were predicted with an accuracy of 0.99 while the Oldenburger animals were all misclassified (0.00). In the OVA scheme, the accuracy reached 0.54, which was only slightly better than random guessing. These results were also reflected by the scaled Brier scores, for which the Oldenburger animals reached a value of $-$0.43, which again was worse than assigning equal probabilities for all classes. The sBS for the other breeds ranged from 0.66 for the Trakehner breed to 0.97 for the Holsteiner, resulting in an overall sBS of 0.58. The SVM performed similar to the OVA Ridge Regression in that scenario, with the main difference lying in the prediction accuracy of the Trakehner animals. The SVM achieved an accuracy of 0.59 in contrast to that of 0.86 for the threshold model.

Generally, the prediction accuracies and scaled Brier scores were below average for the least represented breed in the data set (Trakehner), irrespective of the classifier used. Unbalanced sample sizes in the different classes can cause unreliable probability/classification estimates in weakly represented classes [40]. This is true for training and prediction. We are aware of this issue but addressing it was beyond the scope of the present investigation.

**Fig. 4:**
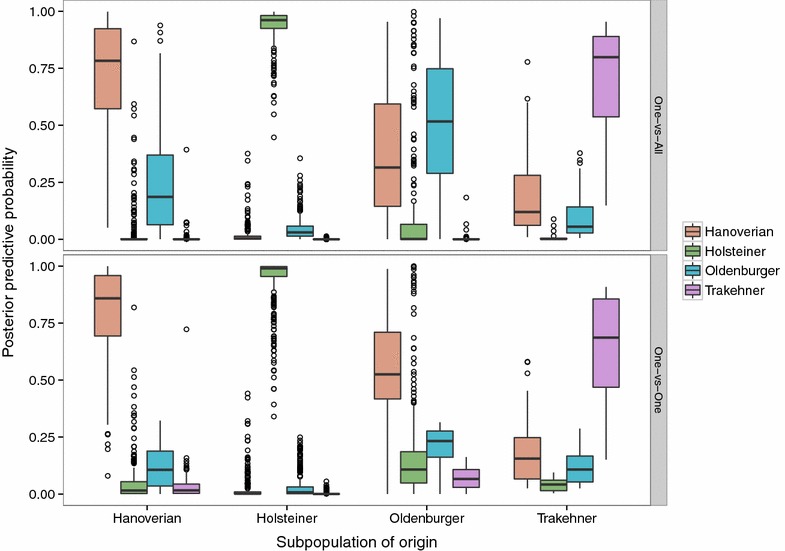
Posterior predictive probabilities by subpopulation and contrast scheme. The binary classifier was a Ridge Regression threshold model. Posterior predictive probabilities for subpopulation assignment were obtained from leave-one-out cross-validation

**Fig. 5:**
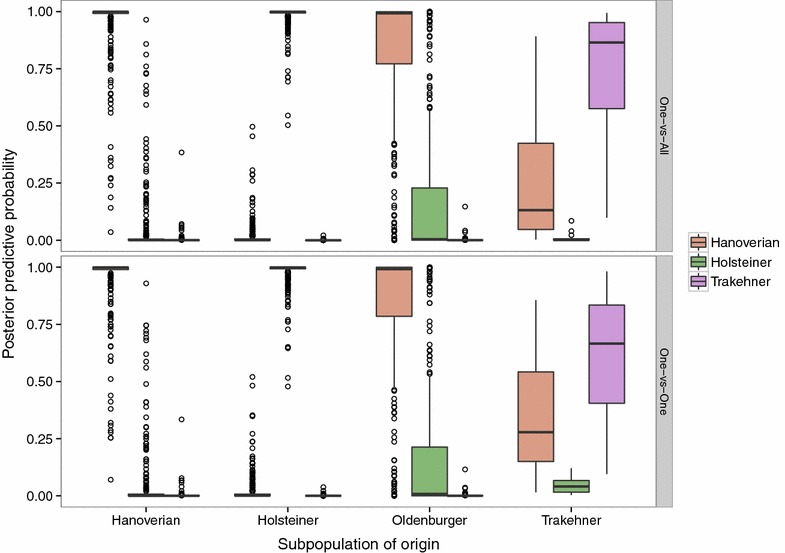
Posterior predictive probabilities by subpopulation and contrast scheme excluding Oldenburger from training. The binary classifier was a Ridge Regression threshold model. Posterior predictive probabilities for subpopulation assignment were obtained from leave-one-out cross-validation

Cross-validation results, when one of the breeds was excluded, provided additional insights into the structure of the data. The schemes that achieved the highest overall and individual accuracies and sBS were those in which either the Hanoverian or Oldenburger breed was excluded. The OVA Ridge Regression model in which all but the Oldenburger breed reached the highest overall accuracies i.e., 0.97, 1.00 and 0.80 for the Hanoverian, Holsteiner and Trakehner breeds, respectively, and the average overall accuracy was equal to 0.97. The results were similar for the scheme that excludes the Hanoverian breed. This result implies that the inclusion of two breeds, even if they are strongly admixed, but given discrete class labels, significantly reduces the power of any of the binary classifiers to unambiguously predict unobserved data to the available classes. The reason is the error in the phenotype measurement, as previously discussed, which gives the class labels. One consequence of this finding is that given the present sample of individuals, the classification of the Hanoverian and Oldenburger animals into two distinct breeds is questionable. Numerous admixed individuals, resulting from stallion exchange between the two breeding organizations, have a strong impact on the results. This becomes more apparent when summarizing the predictive distributions of class label probabilities by subpopulations. This has been done in Figs. 4 and 5. The former visualizes the predicted probabilities from leave-one-out cross-validation for all four subpopulations arranged by breed origin and contrast scheme.

In terms of binary classification, the probability mass concentrated in the class of origin of a particular breed that is higher than 0.5 is of importance. The probabilities in the other classes clearly show a large proportion of individuals that are not unambiguously classified in one or the other class. This was especially true for the Hanoverian and Oldenburger animals. In the OVO scheme, the majority of the Oldenburger individuals had a predicted probability to belong to the Hanoverian breed higher than 0.5, which explains the 0.00 prediction accuracy for that breed and scheme (Table 3). In the OVA scheme, the Oldenburger individuals had on average the highest probability in their own class, although they show substantial probability mass in the remaining classes.

The difference in identical classifiers between the two coupling schemes (OVO vs OVA) used raises the question which one might be more appropriate. Across all scenarios, the OVA Ridge Regression scheme yielded higher accuracies within and between breeds compared to OVO. The leave-one-out scheme appears to be more sensitive to weak contrasts, which is readily explained by the fact that the classifier in that scheme never sees more than two classes. If that particular contrast is weak, the resulting prediction cannot be expected to yield high accuracies. In the OVA scheme, the classifier is always presented as the contrast between one class and all the remaining classes. Hence, even if two classes are not easily separated, the remaining classes still contribute to the binary discrimination. Although the SVM uses the OVO coupling scheme, the prediction accuracies are similar to those obtained with the threshold model using OVA. This might be due to the voting used in contrast to the estimation of posterior predictive probabilities.

In addition to the admixture between the Hanoverian and Oldenburger animals, Fig. 4 shows the strong discriminative ability of the threshold models for the Holsteiner and Trakehner animals. In order to assess the influence of the Oldenburger individuals on the total predictive ability of the threshold model and the admixture with the other breeds, we excluded them from training in Fig. 5. This resulted in significantly improved prediction accuracies for the Hanoverian animals. The prediction model based on Hanoverian, Holsteiner and Trakehner animals only was also used to predict the left out Oldenburger individuals. More then 75 % of them had a predicted probability of belonging to the Hanoverian breed close to 0.8, 50 % of which beyond 0.95. Only a minority of individuals had the highest predicted probabilities for the Holsteiner breed, which reflects the influence of that breed on parts of the sample.

Generally, the classification of unobserved individuals to one (or partially several) subpopulation(s) achieved high cross-validation accuracies. One application would be to use a reference population, that reflects subpopulations or breeds with high confidence, to estimate marker effects. To which subpopulations belong uncertain individuals can be predicted based on the training of those effects if prior breed assignment is doubtful. Probabilities for the different categories can be used to make inference about the genetic background of individuals and assign them to one or partially to several subpopulations under the restriction that the subpopulations represented in the training set are the only choices for classification.

It should be noted that it is certainly possible to increase the overall prediction accuracies by alternative clustering of the data. However, the scope of our investigation was to evaluate the ability to predict unobserved individuals given the original subpopulation assignments. Migration of animals through the exchange of sires has a large impact on the results, but from a breeding organisation perspective the definition of a particular breed still makes sense, even with strong admixture with other populations. Breeding organisations define breeding goals and provide tools that might be more or less restrictive to achieve these goals. Breeding goals are an aggregate of desired phenotypic expressions that the breeders want to achieve by artificial selection and controlled mating. Given an unrestricted breeding policy, breeders would use stallions and mares, regardless of their origin, that most suitably contribute to the given breeding goal, hence having high estimated breeding values for traits of interest.

A recent study investigated the impact of selection according to identical criteria in two distinct populations on the change in allele frequencies of SNPs on a genome-wide scale [41]. The main finding was, that genomic regions that responded to selection by changes in allele frequencies that were greater than those due to drift, did not overlap between populations. The authors reasoned that response of genomic regions to selection depends on the initial allele frequencies in the population. In horse populations, such an investigation is hardly possible considering the long generation intervals. However, after a sufficiently long period of concurrent selection, distinct populations will likely show similar allele frequencies at QTL. Given the available data, it is not possible to distinguish between demographic history and selection but the phenotype “subpopulation assignment” certainly reflects both. An interesting follow-up study would be to investigate the genetic covariance of certain traits under selection between all breeds. Such an analysis would allow the estimation of the ratio of among-population genetic variance ($\sigma _{G}^2$) with total genetic variance, which is the sum of $\sigma _{G}^2$ and the within-population genetic variance ($\sigma _{GW}^2$), i.e. $Q_{ST} = \frac{\sigma _{G}^2}{\sigma _{G}^2+\sigma _{GW}^2}$ [42]. Instead of differentiating between subpopulations using anonymous markers with unknown effects on quantitative traits, one would obtain measures of population divergence based on traits of interest that are under selection. This question could be investigated by an interaction model in which a baseline effect is fitted across breeds while allowing for breed-specific deviations, as proposed by de los Campos and Sorensen [43].

### Genomic prediction of unordered categorical traits

The described methodology for conducting multinomial prediction rests on the assumption that the multiple class problem can be represented by a number of pair-wise binary contrasts. In order to compute posterior predictive probabilities for all classes, it is sufficient to normalize the posterior predictive probabilities from the binary models in the OVA scheme and to solve a fixed point equation in OVO. Besides its appealing simplicity and the ability to make use of the variety of whole-genome regression models used in the genomic prediction framework, it also allows the use of existing software implementations without explicit multinomial extensions. The framework described is not limited to the application given here. There are numerous categorical traits of interest in livestock breeding, that cannot, or only barely, be ordered in a sensitive way to allow for ordinal threshold models to apply. Examples include coat color, functional traits or anatomical discrete measurements. Traits that are usually being ordered like calving ease might also benefit from a non-ordinal treatment of the different classes if the applied ordering scheme is questionable.

#### Quantitative genetic considerations

The binary classification scheme used here implicitly assumes independence between any two contrasts and ignores unobserved classes (independence of irrelevant alternatives, [44]). In the OVA scheme, each class of a categorical trait is treated as an independent binary trait for which exclusive marker effects are estimated.

Defining a single additive genetic variance for unordered categorical traits is counterintuitive and, therefore treating the different categories as potentially correlated traits could provide insights about the genetic architecture of the multivariate trait aggregate. Genetic correlations could be estimated on the liability scales and the multinomial probit model would be a good candidate to investigate this topic since it does not rely on the independence of irrelevant alternatives assumption and can therefore take genetic covariances across the pair-wise binary contrasts into account [44].

In addition, selection index theory could be used to generate a genotypic aggregate of the different categories of an unordered categorical trait. Selection could then proceed on an economically weighted index, since it is very likely that different categories have different economical impacts. The heritability for a single unordered categorical trait would then be defined by the applied index.

#### Alternative prediction of subpopulation assignment

The application given here certainly has alternatives such as substituting the marker matrix used for prediction by the lower Cholesky factor of the numerator relationship matrix ($\mathbf {A}$), resulting in a regression over the pedigree rather than the markers. This has been investigated for the prediction between pairs of breeds. For the threshold model, the marker regression achieved between 5 and 10 % higher prediction accuracies, while the SVM failed to discriminate when the lower Cholesky factor of $\mathbf {A}$ was presented as feature matrix. It was also observed that, when the same factorization of the genomic relationship matrix was used as feature matrix in a SVM, predictions were in the range of those presented in Table 3 but still clearly inferior. This might be due to the fact that the SVM has less features to work with.

An alternative approach for the classification of populations is to use the conditional genotype probabilities of an individual given the estimated allele frequencies in the populations under HWE assumptions [45].

## Conclusions

Multinomial classification using whole-genome regression methods can be assessed by pair-wise binary contrasts in conjunction with appropriate classification rules. The application to the prediction of population assignments revealed high prediction accuracies over all classes except for one. Results of the regression models using an OVA scheme were comparable to the prediction accuracies achieved by support vector machines in an OVO scheme.

The estimated inclusion proportion of eigenvectors (*pEV*) of a genomic relationship matrix in pair-wise contrasts serves as a sensitive indicator for population divergence and can be easily extended for the analysis of quantitative traits, when the number and location of relevant eigenvectors to explain variation in the response are doubtful [21].

The reduction of breeding populations to a single class label does not allow inferences about the divergence of certain quantitative traits between populations. Further research is necessary to determine the genetic variability across the Warmblood horse populations with respect to the traits under selection.
